# Motor Learning Based on Oscillatory Brain Activity Using Transcranial Alternating Current Stimulation: A Review

**DOI:** 10.3390/brainsci11081095

**Published:** 2021-08-20

**Authors:** Naoyuki Takeuchi, Shin-Ichi Izumi

**Affiliations:** 1 Department of Physical Therapy, Akita University Graduate School of Health Sciences 1-1-1, Hondo, Akita 010-8543, Japan; 2 Department of Physical Medicine and Rehabilitation, Tohoku University Graduate School of Medicine, Sendai 980-8575, Japan; izumis@med.tohoku.ac.jp

**Keywords:** oscillatory brain activity, brain communication, transcranial alternating current stimulation, stroke, Parkinson’s disease

## Abstract

Developing effective tools and strategies to promote motor learning is a high-priority scientific and clinical goal. In particular, motor-related areas have been investigated as potential targets to facilitate motor learning by noninvasive brain stimulation (NIBS). In addition to shedding light on the relationship between motor function and oscillatory brain activity, transcranial alternating current stimulation (tACS), which can noninvasively entrain oscillatory brain activity and modulate oscillatory brain communication, has attracted attention as a possible technique to promote motor learning. This review focuses on the use of tACS to enhance motor learning through the manipulation of oscillatory brain activity and its potential clinical applications. We discuss a potential tACS–based approach to ameliorate motor deficits by correcting abnormal oscillatory brain activity and promoting appropriate oscillatory communication in patients after stroke or with Parkinson’s disease. Interpersonal tACS approaches to manipulate intra- and inter-brain communication may result in pro-social effects and could promote the teaching–learning process during rehabilitation sessions with a therapist. The approach of re-establishing oscillatory brain communication through tACS could be effective for motor recovery and might eventually drive the design of new neurorehabilitation approaches based on motor learning.

## 1. Introduction

Motor learning plays a central role in the acquisition of novel actions in various settings, including occupational, sports, music, and rehabilitation activities [1,2,3,4]. Therefore, developing effective tools and strategies to promote motor learning is a high-priority scientific and clinical goal. Multiple brain regions, including the primary motor cortex (M1), premotor cortex, supplementary motor area, and cerebellar cortex, are involved in motor learning [5,6,7]. These brain regions have been investigated as potential targets to facilitate motor learning by noninvasive brain stimulation (NIBS), which can alter cortical excitability through approaches such as repetitive transcranial magnetic stimulation and transcranial direct current stimulation [8,9]. In particular, transcranial alternating current stimulation (tACS), which can non-invasively modulate oscillatory brain activity, has attracted attention as a promising technique to promote motor learning. Neurophysiological studies using electroencephalogram (EEG) and magnetoencephalogram (MEG) have demonstrated that brain oscillatory frequencies, such as alpha (8–13 Hz), beta (13–30 Hz), and gamma bands (30–100 Hz), are associated with motor control and learning [10,11,12,13,14]. tACS is a non-invasive electrical stimulation that applies a weak oscillatory current to the brain through the scalp to entrain neuronal activity into these frequency patterns [15,16,17]. tACS induces the neural membrane potential to oscillate away from its resting potential towards slightly more depolarized or hyperpolarized states. Neurons currently in a depolarization state are more likely to fire in response to other neurons. This is referred to as “stochastic resonance” and is thought to be one possible mechanism by which tACS can entrain neural activity into the stimulated frequency [16,18,19]. The brain’s complex processes depend on coordinated communication among large-scale distributed brain networks. Flexible and rapid information transfer across distant brain areas is necessary to support these functions [20,21]. As a mechanism of the human brain’s complex processing capabilities, synchronization of oscillatory brain activity between distant cortical areas is hypothesized to facilitate information transfer by temporally aligning neural processing across brain areas [21,22]. Therefore, concurrent tACS over distant cortical regions can effectively modulate oscillatory phase synchrony and functional connectivity between the targeted brain regions by entraining brain oscillations [15,23,24]. Several studies have demonstrated that the synchronization of oscillatory activity on multiple temporal scales across brain regions is associated with motor function in humans [25,26,27,28]. There is accumulating evidence that abnormal oscillatory brain activity and changes in oscillatory communication within and between motor-related regions are associated with motor deficits in patients with stroke and Parkinson’s disease (PD) [29,30,31,32]. Therefore, tACS has emerged as a promising therapeutic approach for ameliorating motor deficits by non-invasively modulating oscillatory brain activity and communication via entrainment of specific frequency oscillations [33,34]. Despite its clinical potential, tACS studies aimed at ameliorating motor deficits in patients with stroke and PD remain scarce.

In this review, we explore the possibility of using tACS to improve motor function in a clinical setting as follows:

(1) We overview motor learning studies using tACS over a single brain region.
(2) We discuss the tACS approach targeting multiple concurrent brain regions to enhance motor learning by manipulating oscillatory brain communication.
(3) We review the abnormal oscillatory brain activity and communication associated with motor deficits in patients with stroke and PD, and then discuss the potential of tACS to ameliorate behavioral deficits by correcting abnormal oscillatory brain activity and promoting appropriate oscillatory communication.
(4) We discuss the future of tACS, which may harness novel approaches such as personalized stimulation parameters and dual brain stimulation, considering interpersonal interactions to stabilize and facilitate motor learning processes.

We present a new, promising tACS approach aimed at promoting motor learning, consisting of physiologically motivated protocols based on oscillatory activity.

## 2. Motor Learning by tACS over Single Site

In addition to brain oscillation entrainment, spike-timing-dependent plasticity has been reported as a tACS effect, but it remains unknown how this aftereffect modulates motor function [24,35]. Therefore, we provide an overview of the role of tACS in motor learning in terms of its capacity to entrain specific frequencies related to motor function. Moreover, the stimulation conditions vary broadly across the literature; therefore, we discuss this issue by classifying the studies into two major categories: stimulation sites (M1, non-primary motor cortex, and cerebellum) and stimulation frequency (alpha, beta, and gamma bands).

### 2.1. Primary Motor Cortex (M1)

Several studies have shown that alpha-tACS over M1 before or during training enhances motor skill acquisition [36,37,38] (Table 1). However, other studies have reported that alpha-tACS over M1 before training has no effect on motor skill acquisition [39,40]. Moreover, alpha-tACS over M1 after training may be detrimental to motor skill consolidation in older adults [41]. The relationship between the alpha rhythm and inhibitory function may be a possible reason for the negative effect of alpha-tACS on motor learning [42,43]. Excessive inhibition within the brain has been reported to reduce neural plasticity [44]. Therefore, enhancing alpha-band oscillatory activity by post-training alpha-tACS may block the consolidation of motor sequence learning. Fresnoza et al. reported that the negative effect of alpha-tACS depends on age. Both an individual’s alpha-tACS and an individual’s alpha + 2-Hz tACS after the first training session facilitated motor skill consolidation in post-tACS training sessions in older adults, while an individual’s alpha + 2-Hz tACS had a negative effect on motor skill consolidation in young subjects [45]. Thus, the effect of alpha-tACS on motor learning depends on several factors, such as timing between stimulation and training, age, and an individual’s alpha frequency.

Beta-band oscillations in M1 are related to motor learning, imagery, and execution [13,14]. It has been reported that beta-tACS over M1 during training enhances motor skill acquisition [37], and that beta-tACS after training facilitates retrieval of motor sequences in the early phase of the post-tACS training session [39]. However, other studies have shown that beta-tACS before [38,40], during [36], and after training [41,49] has no effect on motor skill acquisition or consolidation. In another study, beta-tACS during training had a detrimental effect on motor skill acquisition during stimulation, but there was no difference in motor skill retention after tACS between beta-tACS and sham stimulation [47]. This negative effect during beta-tACS appears to be consistent with previous reports that motor performance deteriorates during beta-tACS [50,51]. A meta-analysis has shown that beta-tACS over M1 is able to increase corticospinal excitability with a small-to-moderate effect size in healthy volunteers [52]; therefore, this excitatory effect might ameliorate the deterioration of motor performance during beta-tACS.

Gamma-band activity in M1 is known to increase during motor preparation and execution [10,11]. Consistent with these physiological findings, high-gamma (70 Hz) tACS over M1 before training was found to enhance motor skill acquisition [40]. However, high-gamma tACS over M1 during training has a negative effect on motor skill retention, although it slightly improves the acceleration of the practiced movement during the initial training phase [47]. Previous studies have also reported that motor functions such as finger movement velocity [53] and force [50] are enhanced during gamma-tACS over M1. Taken together, these results indicate that the temporary enhancement of motor function during gamma-tACS does not necessarily lead to the consolidation of motor learning. To summarize, offline administration of high-gamma tACS over M1 before training may be more suitable for motor learning than that in online stimulation during training. Online low-gamma (<45 Hz) tACS over M1 also seems to be ineffective for motor learning [36,37,46].

The heterogeneous effect of uniform frequency tACS over M1 on motor learning might be due to different stimulation parameters (intensity, frequency, duration, electrode size, and electrode position), various tasks, and great variability among tACS responders and non-responders. Specifically, given that the position and size of the electrodes have a significant influence on current flow in the brain, the focal stimulation technique is needed to reduce the variability of the tACS effect, as discussed later in Section 5.1. As a promising tACS parameter, a recent study has reported that gamma and theta phase-amplitude coupling (PAC) tACS over M1 during training promotes motor skill acquisition [48]. Theta–gamma PAC, which appears to be a common phenomenon across the cortex, has been hypothesized to be a fundamental operation of cortical computation in neocortical areas [54]. Therefore, the tACS protocol using theta-gamma PAC may be more effective for motor learning than that in tACS with a uniform frequency.

### 2.2. Non-Primary Motor Cortex and Cerebellum

There are few studies on motor learning using tACS over brain areas other than M1 (Table 2). One study found that an individual’s alpha-tACS over the premotor cortex before training was effective for motor skill acquisition [55]. Although not an assessment of motor learning, Hsu et al. investigated whether tACS over the premotor cortex altered multitasking performance [56]. It is well established that the premotor cortex is involved in multitasking [57,58]. Multitasking performance improved when the bilateral premotor cortices were stimulated by theta (6 Hz) tACS during a task. Therefore, tACS over the premotor cortex is a potential approach for motor learning related to multitasking. The supplementary motor area is an important cortical area for motor planning and execution of sequential and continuous bimanual movement tasks [59,60]. Miyaguchi et al. found that bilateral movements improved when tACS was administered to the supplementary motor area during a task. Beta-tACS showed a significant correlation with improved motor skill acquisition in subjects with higher initial motor performance, while gamma-tACS showed a significant correlation with improved motor skill acquisition in subjects with lower initial motor performance [61]. The cerebellum is considered a core node of the network related to motor learning [5]. Therefore, the cerebellum is expected to be a target of tACS to promote motor learning. Naro et al. have reported that gamma-tACS over the cerebellar cortex enhances motor skill acquisition immediately after tACS [62]. However, a recent study has reported that gamma-tACS over the cerebellar cortex during a sequential grip force task does not enhance motor skill acquisition or retention [63]. Thus, while the number of studies using tACS is gradually increasing, to date, there are few studies reporting on the effect of tACS over the non-primary motor cortex and cerebellum on motor learning compared with M1.

## 3. Motor Learning by tACS Targeting Brain Communication

**Figure 1:**
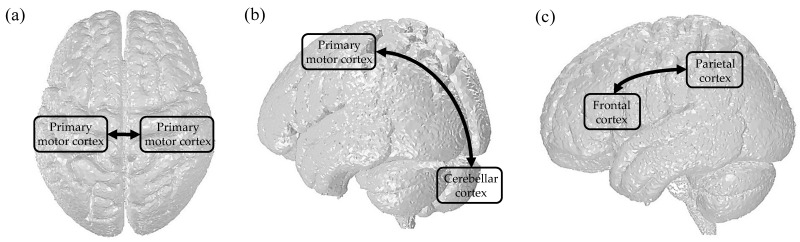
tACS targeting brain communication. (**a**) between bilateral primary motor cortices. (**b**) between primary motor cortex and cerebellar cortex. (**c**) between frontal and parietal cortices.

There is mounting evidence that several brain networks are associated with motor learning [6,64,65,66]. Among them, neural communications in the bilateral M1s and the M1-cerebellum are strongly involved in the process of motor learning. In addition, it has been speculated that the frontal and parietal regions might work together to participate in the imitation process, which is important for the acquisition of new behaviors. In this section, we discuss the tACS protocol for motor learning through exogenous synchronization of oscillatory brain activity across distant areas (Figure 1).

### 3.1. Communication between Bilateral M1s

It is well established that the effect of unilateral motor training transfers to the contralateral hand in motor paradigms such as serial reaction time tasks [67] and sequential pinch force tasks [65]. This phenomenon is called intermanual transfer or cross-training effect [65,68]. Although the underlying neural mechanisms of intermanual transfer remain elusive, there is evidence that plasticity within the M1 ipsilateral to the trained hand might play an important role in mediating performance improvements in the untrained hand [69,70]. Therefore, the interhemispheric interaction between the homologous M1s via the corpus callosum could be a potential mechanism mediating intermanual transfer [28,65,67]. Takeuchi et al. found that synchronous beta-tACS over the bilateral M1s during the mirror drawing task improved performance of the same task in the untrained hand immediately after stimulation, while sham and gamma-tACS did not affect it [71]. Consistent with these findings, EEG studies have revealed that an increase in interhemispheric beta-band coherence is associated with intermanual transfer [28,72]. The beta-band synchronous neural oscillation induced by tACS in both M1s might increase the long-range interhemispheric information circulating from the trained M1 to the untrained M1, resulting in the enhancement of intermanual transfer.

The interhemispheric interaction between the M1s also plays a pivotal role in the coordination of bimanual movements. Heise et al. applied synchronous tACS over the bilateral M1s concurrently to a bimanual coordination task. While beta-tACS negatively impacted bimanual coordination, alpha-tACS improved it [73]. However, another study found that synchronous tACS over the bilateral M1s increased errors in bilateral coordination tasks at both 10 Hz and 20 Hz [74]. While these studies used different tasks, they suggest that the interhemispheric synchronization induced by tACS over the bilateral M1s might deteriorate the flexible control of complex bimanual actions by strengthening interhemispheric inhibitory interactions [75]. In patients with stroke and/or musculoskeletal injury, strengthening the interhemispheric network efficiency between the bilateral M1s by synchronous beta-tACS might thus be a promising approach to improving the function of the affected limb by training the healthy limb. However, it might also have a negative effect on motor skills that require complex bimanual coordination.

### 3.2. Communication between M1 and Cerebellar Cortex

The neural network of the M1–cerebellum is known to be important in the early motor learning phase [64,66]. Both M1 and the cerebellar cortex play essential roles in motor skill retention [5,76]. Therefore, if the brain network of the M1–cerebellum can be strengthened by tACS, motor learning may be enhanced. This hypothesis has been supported by a recent study showing that gamma-tACS administered over the M1–cerebellum during training can enhance motor skill retention [26]. Gamma-band activity in the cerebellum is important for the synchronous activity of sensorimotor areas [27]. Therefore, the M1–cerebellum brain communication facilitated by gamma-tACS results in enhanced motor skill retention. Of note, in the study, motor learning improved in the anti-phase condition, not in the in-phase [26]. It has been speculated that it takes approximately 5–7 ms for neurotransmission between the M1 and the cerebellar cortex to occur [77]. The time required for one cycle of the alternating current waveform when stimulated at a frequency of 70 Hz is approximately 14 ms. Thus, in the anti-phase condition, when the signal from the cerebellar cortex area was transmitted to M1, taking approximately 5–7 ms, the current flowing to the electrode placed on M1 may have been in phase with the current flowing in the cerebellar cortex. Therefore, anti-phase gamma-tACS may have strengthened the functional synchronization between M1 and the cerebellum, resulting in the enhancement of motor learning [26]. As discussed in the previous section focusing on a single site, gamma-tACS over the cerebellar cortex only is insufficient for enhancing motor learning. Stimulation over more than one region, to take into account brain communication in the cerebellar cortex, might be desirable for motor learning.

### 3.3. Communication between Frontal and Parietal Cortices

Imitation implies learning and requires the integration of visual information with the motor representation of an action [78,79]. Therefore, it has been speculated that the imitation process is controlled by the frontoparietal mirror network, which is activated when an individual performs a goal-directed action in a process similar to when they observe another person performing the same action [80,81,82,83]. Takeuchi et al. demonstrated that synchronous theta–gamma PAC tACS over the left inferior frontal gyrus and the left inferior parietal lobule improved meaningless gesture imitation [84]. In contrast, desynchronous tACS caused a deterioration of performance in the gesture-matching task relative to baseline performance. These results indicate that the increased rhythmic, in-phase synchrony between components of the left frontoparietal mirror network induced by synchronous tACS facilitates the imitation process by strengthening network efficiency. Imitation ability is a very important factor in rehabilitation. In particular, patients with apraxia whose imitation performance is impaired are less likely to improve their daily life activities after rehabilitation and more likely have a persistent motor learning deficit [85,86]. Therefore, the potential of synchronous tACS to strengthen the network efficiency of the frontoparietal mirror network might be a promising approach to enhance rehabilitation based on motor learning in patients with apraxia. However, the role of the network between the frontal and parietal lobes in motor learning is controversial. A study reported that beta-band resting-state functional connectivity between M1 and the parietal area is positively associated with motor skill acquisition [87]. In contrast, another study found that beta-band resting-state functional connectivity in the M1–parietal area was negatively correlated with motor skill acquisition [66]. Therefore, future tACS studies must evaluate the causal role of M1–parietal brain communication in motor learning.

Synchronous oscillatory activity is thought to represent the basic mechanism of functional communication [88,89]. Therefore, synchronous tACS over distant areas is generally used to strengthen brain communication. However, it might be necessary to adjust the phase of tACS by considering the transmission time between the distant targeted areas and the stimulus frequency. A recent theoretical model shows that bi-directional communication at low frequencies is due to zero-phase synchronization, but relatively fast frequencies, such as the gamma-band synchronization, entail a non-zero phase lag [22,90]. In summary, synchronized stimulation by tACS may be appropriate for inter-cerebrum communication with the view of promoting motor learning, but phase-shifted stimulation must consider the transmission time for cerebrum-cerebellum communication.

## 4. tACS Approach for Motor Deficits in Stroke and PD Patients

There is mounting evidence that changes in oscillatory brain activity and communication within and between motor-related regions are associated with motor deficits in patients with stroke and PD [29,30,31,32]. In this section, we provide an overview of studies investigating oscillatory brain activity after stroke and in PD, and then discuss the clinical potential of tACS to improve motor function through the correction of abnormal brain oscillations and the promotion of appropriate oscillatory brain communication.

### 4.1. Stroke

It is well established that brain oscillations change after stroke [91,92]. Increased slow rhythms and decreased fast rhythms are directly linked to neuronal metabolism, which reflects stroke injury [29,93,94]. Therefore, abnormal oscillatory activities near stroke lesions might not be suitable targets for tACS, because they may merely represent metabolic changes induced by stroke injury. Although the extent of corticospinal tract integrity is strongly correlated with motor recovery after stroke [95], the change in brain communication across motor-related regions has also been shown to be paralleled by motor recovery after stroke using brain imaging techniques [96,97]. EEG/MEG studies have also demonstrated that changes in oscillatory brain communication are associated with motor recovery. Westlake et al. reported that greater alpha-band coherence of the ipsilesional sensorimotor cortex and prefrontal cortex with the entire brain was correlated with better motor recovery after 8–12 weeks in stroke patients. In contrast, the lower the alpha-band coherence of the contralesional sensorimotor regions with the entire brain, the better the motor recovery [98]. Wu et al. reported that the beta-band coherence of the ipsilesional M1-premotor was positively correlated with motor function in patients with chronic stroke. Moreover, beta-band coherence of the ipsilesional M1-premotor increased in parallel with greater motor gains after rehabilitation [30]. Beta-band coherence between the bilateral M1s was positively correlated with upper limb motor function in patients with subacute and chronic stroke [99]. Thus, the increase in the brain communication centered in the ipsilesional hemisphere after stroke may be beneficial for motor recovery, whereas the brain communication taking place in the contralateral hemisphere may be maladaptive. However, not all brain communication related to the ipsilesional hemisphere is beneficial for motor recovery. Nicolo et al. have reported that the increase in beta-band coherence between the ipsilesional M1 and all other brain regions negatively correlates with motor recovery in patients with chronic stroke [29]. Moreover, the reduction of beta-band coherence between the ipsilesional M1 and parietal lobe was associated with greater motor gains in patients with chronic stroke [30]. A range of individual factors including stroke lesion, time since stroke onset, severity of motor impairment, and compensation of impairments might lead to high inter-subject variability in the changes in brain oscillatory communication.

While inter-individual variability should be considered, re-establishing oscillatory brain communication through tACS might be effective for motor recovery and could eventually drive the design of new rehabilitation approaches based on oscillatory activity. Consistent with this hypothesis, sensorimotor rhythm modulation training of alpha-band coherence between the ipsilesional M1 and the rest of the brain using EEG neurofeedback enhanced motor performance in patients with chronic stroke [100]. To date, studies have reported the clinical possibilities of using tACS as an adjunct to a brain-computer interface, combining gait-synchronized tACS with neuromuscular stimulation, and inducing changes in brain communication after tACS; however there have been no tACS studies in which the motor recovery after stroke was facilitated by modulating oscillatory brain communication. Naros et al. reported that beta-tACS over the ipsilesional M1 facilitated the classification accuracy of the brain–computer interface based on beta-desynchronization during motor imagery in patients with chronic stroke. It is known that movement-related beta-desynchronization is less pronounced in stroke patients with severe motor impairment [91]. Therefore, beta-tACS may constitute an adjunct neuromodulation technique for neurofeedback-based interventions for stroke rehabilitation by enhancing beta-desynchronization [101]. Koganemaru et al. reported that gait-synchronized tACS over the ipsilesional M1 combined with neuromuscular stimulation over the paretic tibialis anterior muscle improved walking speed in patients with chronic stroke [102]. While tACS synchronized with movement may be effective for motor recovery, tACS has been shown to be strongly affected by scalp sensory stimulation [103]. Therefore, an appropriate tACS control setting over a site other than a motor-related area is necessary to rule out the possibility that the positive effect on motor function is merely derived from movement-synchronized peripheral stimulation. Chen et al. reported that beta-tACS over the ipsilesional M1 facilitated local segregation in motor-related regions and global integration at the whole-brain level in patients with chronic stroke. However, alpha-tACS was only observed to increase segregation at the whole-brain level [104]. The authors suggested that beta-tACS over the ipsilesional M1 has the potential to facilitate neurorehabilitation for motor recovery because it might induce more modulation effects in motor-related regions. Altogether, there are few studies on stroke using tACS, and further investigation is needed.

### 4.2. PD

In PD patients, there is excessive beta-band activity in the cortex–basal ganglia network [105,106,107,108]. A relationship between abnormal beta-band oscillations and PD symptoms has also been reported. The beta-band increase in the cortex–basal ganglia network correlates with bradykinesia [108,109], rigidity [110], and freezing of gait (FOG) [111]. Moreover, beta-band coherence between the supplemental motor area and M1 was stronger in PD patients with FOG than in PD patients without FOG [32]. In contrast, the gamma band in the cortex–basal ganglia network has been shown to decrease in PD off-medication and to increase with dopaminergic medication, correlating with clinical improvement [112,113]. Thus, beta-frequency excess in the cortex–basal ganglia network seems to have an anti-kinetic effect, while gamma-band activity seems to have a pro-kinetic effect in patients with PD. Therefore, it can be hypothesized that attenuation of beta-band and enhancement of gamma-band activities by tACS over the cerebral cortex may alleviate PD symptoms by modulating the entire cortex–basal ganglia network through cortico-subcortical loops. However, only a handful of studies have applied tACS to relieve PD symptoms.

Brittain et al. demonstrated that tACS suppressed resting tremor in patients with PD by applying a phase cancelation technique. By delivering tremor-frequency tACS over M1, they identified the timing of the greatest change in tremor amplitude during slow alternating periods of phase cancelation and reinforcement. tACS delivered at the same timing as the tremor cancelation phase can achieve an average reduction of almost 50% in the resting tremor amplitude [114]. Another exploratory study showed that tremor-frequency tACS over the cerebellar cortex had no effect on tremor amplitude [115]. De Felice et al. evaluated whether personalized tACS combined with physical therapy improved motor performance in patients with PD. The electrode position of tACS was individually defined based on a statistical comparison of EEG power spectra maps. The stimulation frequency was also set according to the EEG band displaying higher power spectra (for beta excess on EEG map, tACS was set at 4 Hz; for theta excess, tACS was set at 30 Hz). Personalized tACS in the 4 Hz group improved motor performance in PD patients, and these improvements were associated with a reduction in excessive beta-band power [19]. A recent study demonstrated that co-stimulation of M1 combined with gamma-tACS and intermittent theta-burst stimulation (iTBS) improved the long-term potentiation (LTP)-like plasticity induced by iTBS in patients with PD [116]. It has been reported that the LTP-like plasticity of M1 is impaired in PD [117]. These findings suggest that gamma-tACS normalizes the cortical gamma oscillations that are altered in the cortex–basal ganglia network in PD, resulting in restoration of the impaired LTP-like plasticity of M1. Therefore, co-stimulation combined with gamma-tACS and iTBS may promote motor learning by restoring neural plasticity in patients with PD.

The concept that PD symptoms are related to dysfunction in the multilevel, interconnected complex cortex–basal ganglia network, rather than the basal ganglia only, has opened up the possibility of modifying these networks by NIBS [34]. However, the application of tACS to PD symptoms has remained experimental, and a systematic review concluded there was no evidence supporting the treatment of PD using tACS [33]. It was found that not only the uniform-band frequency but also the excessive beta-gamma PAC at M1 played an important role in the pathophysiology of PD [31]. The normalization of abnormal beta-gamma PAC using tACS is expected to be a future therapeutic target for PD.

## 5. Future tACS Approaches to Stabilize and Promote Motor Learning

With the increasing knowledge on the relationship between motor deficits and oscillatory brain activity in neurological disorders, clinical approaches using tACS have been gradually investigated. However, inter-individual variability and pathological differences are major obstacles in stabilizing the tACS effect. In this section, with a view to stabilizing the tACS effect and promoting motor learning, we review the necessity of focal tACS and closed-loop tACS according to the brain state, and then discuss concurrent tACS over two individuals’ brains considering interpersonal interaction.

### 5.1. Focal and Personalized tACS for Appropriate Stimulation

Although conventional tACS using two large rubber electrodes (approximately 3–7 cm in diameter or length) is easy to handle, it has the disadvantage that the current flow is not localized, as it flows to the reference electrode. Conventional non-focal electrode placement may be one of the reasons for the inconsistent tACS results. The use of a 4 × 1 ring electrode configuration or computationally optimized multi-channel arrangements can help to focus on the stimulated area [118,119]. The variability of tACS is also strongly influenced by individual anatomical factors, such as gyral folding, cerebrospinal fluid thickness, and skull composition [120]. Moreover, computational model studies suggest that sex-related anatomical differences may affect current flow induced by tACS [121,122]. Therefore, it is important to confirm that the appropriate current flows to the target area using computational models with actual magnetic resonance imaging (MRI) images, especially when treating patients with anatomical brain changes in a clinical setting.

As a new technique, transcranial temporal interference stimulation has been proposed to stimulate deep brain regions with specific frequencies and amplitudes using temporally interfering electric fields [123]. In this multifocal alternating electric current stimulation approach, two slightly different frequencies are delivered in the kHz range, both of which are far beyond the frequencies stimulating pain receptors and the cortical territory. The difference between the two frequencies is the target frequency, because the sum of the two electrical fields appears as an amplitude-modulated signal at the frequency of the difference between the two original frequencies in an area where the electric fields overlap. A new protocol should be developed to promote motor learning by directly stimulating the basal ganglia with this technique. However, it has only been investigated in mice and needs validation of its safety in humans.

In addition to focality, it is important to personalize the stimulation frequency to stabilize and promote the tACS effect. It is thought that the entrainment induced by tACS is more effective when the stimulated frequency matches the ongoing endogenous rhythm [124,125]. However, as in the conventional tACS approach, even if a fixed stimulation frequency is adjusted to the individual’s frequency before tACS, the internal and the stimulation frequencies are not always matched because the brain oscillations fluctuate in frequency and amplitude over time. The frequency of tACS must be constantly adapted to the ongoing internal frequency to optimally adjust the stimulation frequency to the internal frequency. Such a system would represent a closed-loop control system. This closed-loop system, which consists of a high-temporal resolution recording (EEG/MEG) of brain activity combined with tACS, might provide neural activity feedback on the intervention and enable the prediction of individual responses, resulting in maximization of the effect by personalizing the tACS parameters, such as location, frequency, and phase [126,127]. This system might also assist in tackling the phenomenon of cross-frequency coupling, in which a stimulation frequency band modulates another frequency band [128]. Moreover, a closed-loop system that monitors the changes in brain oscillatory activities in real time is expected to be beneficial for appropriate alteration of the tACS parameters to induce the target oscillatory frequency in pathological conditions where changes in oscillatory brain activity induced by tACS may be different from those in healthy subjects. However, it is necessary to analyze brain oscillatory activities in real time and remove the artifacts produced by tACS without taking out large amounts of valuable electrophysiological signals, and no system has been developed to this end yet. The current state of closed-loop applications of tACS techniques has been described elsewhere [127,129].

### 5.2. tACS Considering Interpersonal Interaction

Studies measuring the activity of multiple brains simultaneously, termed “hyperscanning,” have revealed that various aspects of interpersonal interaction can be reflected in inter-brain synchronization between individuals engaging in joint actions, communication, and teaching–learning tasks [130,131,132,133,134]. Based on the findings from hyperscanning studies, the simultaneous application of tACS to two individuals (hyper-tACS) has been developed to modulate inter-brain synchronized oscillation frequencies and artificially operate interpersonal interaction, instead of modulating only one brain.

Novembre et al. first evaluated whether hyper-tACS alters interpersonal movement. In-phase beta-tACS over M1 in pairs of individuals who both performed a finger-tapping task enhanced interpersonal movement synchrony, compared with anti-phase or sham stimulation. Phase coupling of brain oscillations across two individuals’ M1s supports the interpersonal alignment of sensorimotor processes that regulate rhythmic action, thereby facilitating synchronous interpersonal movement [135]. However, another study reported that theta hyper-tACS over the left frontocentral and centroparietal sites during joint action results in impairment, rather than improving the dyadic drumming synchrony task [136]. Although it is possible that the frequency and site of tACS were inappropriate to the promotion of this dyadic task, the frequency entrained by tACS may differ between individuals, resulting in a reduction in dyadic synchrony in the tACS condition.

Interpersonal interaction is also important in rehabilitation situations, such as when a therapist teaches motor skills to a patient. Hyperscanning studies have shown the importance of the prefrontal cortex in the teaching–learning process [137,138], and it has been reported that the neural activities in the prefrontal cortex in both the instructor and the learner become synchronized during the teaching–learning task [134]. Pan et al. studied whether hyper-tACS over the inferior frontal cortex can facilitate the teaching–learning process. In-phase theta hyper-tACS in both the instructor and the learner can augment social interactive learning during a naturalistic song-learning task [139]. Moreover, this hyper-tACS synchronized the body movement between the instructor and the learner. These effects were both phase- and frequency-specific; neither anti-phase theta-tACS or in-phase alpha-tACS yielded comparable results. Interpersonal movement synchrony is known to induce pro-social effects, such as rapport, cooperation, and affiliation [140,141]. These factors might also have a positive impact on the teaching–learning process [142,143].

Dual brain stimulation using hyper-tACS to operate the brain oscillations of not only the patients but also the therapists may not be realistic in rehabilitation situations. It is desirable to promote interpersonal interaction by operating only the patient’s brain oscillations by adjusting tACS to the therapist’s brain oscillations. Of note, oscillatory brain activities in the leader and the follower are not completely synchronized in time and space. Directed coherence has been reported between the activity at the frontal sides in the leader’s brain and the activity at the frontal and parietal sides in the follower’s brain when they play the card game [144]. Therefore, future tACS studies aimed at promoting motor learning related to interpersonal interactions must take into account these temporal and spatial asymmetries in the brain activities between patients and therapists.

## 6. Conclusions

As discussed in this review, tACS tailored to brain oscillations is expected to facilitate motor learning, but to date, there is little direct evidence to support this. To help fill the gap, we discussed the tACS approach from the perspective of oscillatory brain communication, closed-loop systems, and interpersonal interaction. Inter-brain synchronization using tACS might be effective for motor learning by facilitating the teaching–learning process. Moreover, tACS combined with other neurorehabilitation strategies might synergistically promote motor learning. However, when different neurorehabilitation therapies are combined, the timing of each therapeutic program must be considered to enable optimal neural plasticity. Homeostatic metaplasticity, which stabilizes the activity of neurons and neural circuits, can either augment or reduce the synergistic effect, depending on the timing of the combination therapy and the types of neurorehabilitation activities used [145]. The development of a closed-loop system that decodes brain activity might reveal the optimal timing between tACS and neurorehabilitation therapies and facilitate the synergic effect by monitoring intrinsic variations in brain oscillations, which may influence homeostatic metaplasticity. Moreover, it is still unclear whether the changes in oscillatory brain activities have a pathophysiological role, or whether they represent true reorganization and compensatory roles in the behavioral deficits and/or injury, or whether they merely represent the neural changes. Therefore, research aimed at modifying motor deficits by modulating oscillatory activities using tACS will also lead to the elucidation of the causal role of abnormal oscillatory brain activities in motor symptoms of neurological disorders.

## Figures and Tables

**Table 1:** Summary of studies applying tACS over the primary motor cortex (M1).

Authors	Study Design	Motor Training Task	Electrode Position	tACS Parameters	tACS Timing	Behavioral Results
Antal et al., 2008 [36]	Crossover *n* = 16	SSRT	Active: left M1 (4 × 4 cm) Reference: right supraorbital (5 × 10 cm)	1 Hz, 10 Hz, 15 Hz, 45 Hz, sham (0.4 mA, about 7 min)	tACS during motor training	10-Hz tACS facilitated motor acquisition during stimulation, but there was no difference between the 10 Hz and sham groups at 1 h after tACS
Pollok et al., 2015 [37]	Crossover *n* = 13	SSRT	Active: left M1 (5 × 7 cm) Reference: right supraorbital (5 × 7 cm)	10 Hz, 20 Hz, 35 Hz, sham (1 mA, about 12 min)	tACS during motor training	Both 10-Hz and 20-Hz tACS facilitated motor acquisition during stimulation compared to sham and 35-Hz tACS
Krause et al., 2016 [39]	Randomly assigned *n* = 36	SSRT	Active: left M1 (5 × 7 cm) Reference: right supraorbital (5 × 7 cm)	10 Hz, 20 Hz, sham (1 mA, 10 min)	tACS during rest time between the first and second sessions of motor training	20-Hz tACS facilitated retrieval of motor sequence in the early second session of motor training compared with 10-Hz and sham groups. However, there was no difference between tACS and sham at the late phase of the second session
Sugata et al., 2018 [40]	Randomly assigned *n* = 52	SSRT	Active: left M1 (5 × 7 cm) Reference: right supraorbital (5 × 7 cm)	10 Hz, 20 Hz, 70 Hz, sham (1 mA, 10 min)	tACS during rest time between the first and second sessions of motor training	70-Hz tACS improved capacity for motor learning in the second session of motor training compared to sham stimulation
Giustiniani et al., 2019 [46]	Crossover *n* = 17	SSRT	Active: left M1 (5 × 5 cm) Reference: right supraorbital (5 × 5 cm)	1 Hz, 40 Hz, sham (2 mA, about 5 min)	tACS during motor training	40-Hz tACS inhibited motor acquisition compared to sham stimulation
Rumpf et al., 2019 [41]	Crossover *n* = 16 (10 Hz vs. sham) *n* = 17 (20 Hz vs. sham)	SSRT	Active: left M1 (radius 3.75 cm) Reference: right supraorbital (5 × 7 cm)	10 Hz, 20 Hz, sham (1 mA, 15 min)	tACS immediately after motor training	10-Hz tACS disrupted motor consolidation 6 hr after tACS compared to sham stimulation
Bologna et al., 2019 [47]	Crossover *n* = 16	Rapid abduction of index finger task	Active: left M1 (5 × 5 cm) Reference: Pz (5 × 5 cm)	20 Hz, 70 Hz, sham (1 mA, about 15 min)	tACS during motor training	20-Hz tACS had a detrimental effect on motor acquisition during stimulation, but there was no difference in motor retention after stimulation between 20 Hz and sham groups. 70-Hz tACS improved motor acquisition during stimulation, but it had a detrimental effect on motor retention
Akkad et al., 2019 [48]	Randomly assigned *n* = 58	Thumb abduction	Active: right M1 (5 × 5 cm) Reference: Pz (5 × 5 cm)	Theta–gamma peak, theta–gamma trough, sham (75-Hz rhythm was amplitude-modulated by the peak or trough envelope of 6-Hz rhythm) (2 mA, 20 min)	tACS during motor training	Theta–gamma peak tACS improved motor acquisition compared with sham stimulation for 75 min after tACS
Roshchupkina et al., 2020 [49]	Randomly assigned *n* = 62	SSRT	Active: right M1 (5 × 5 cm) Reference: left deltoid (5 × 5 cm)	20 Hz, sham (1 mA, about 10 min)	tACS immediately and 25 min after motor training	20-Hz tACS did not influence early (at 4 hr) and long-term (at 24 hr) motor skill consolidation
Harada et al., 2020 [38]	Randomly assigned *n* = 33	Visuomotor adaptation task	Active: left M1 (5 × 7 cm) Reference: right supraorbital (5 × 7 cm)	10 Hz, 20 Hz, sham(1 mA, 10 min)	tACS before motor training	10-Hz tACS facilitated initial motor acquisition compared with 20-Hz tACS and sham stimulation. However, there was no significant difference in task performance at the late phase among the three groups
Fresnoza et al., 2020 [45]	Crossover n = 20 (young group) *n* = 15 (old group)	SSRT	Active: left M1 (5 × 7 cm) Reference: right supraorbital (5 × 7 cm)	Individual‘s alpha, individual‘s alpha + 2 Hz, sham (1.5 mA, 15 min)	tACS during rest time between the first and three subsequent sessions of motor training (immediately, 60 min, and 120 min after tACS)	Both the individual’s alpha-tACS and the individual’s alpha + 2 Hz-tACS improved consolidation of general motor and sequence-specific skills during post-tACS training sessions in the old group. The individual’s alpha-tACS impaired consolidation of sequence-specific skills and the individual’s alpha + 2 Hz-tACS was detrimental to the consolidation of both skills in the young group

SSRT: serial reaction time task.

**Table 2:** Summary of studies applying tACS over non-primary motor cortex or cerebellum.

Authors	Study Design	Motor Training Task	Electrode Position	tACS Parameters	tACS Timing	Behavioral Results
Naro et al., 2016 [62]	Crossover *n* = 25	Sequential finger tapping	Active: right cerebellar cortex (5 × 5 cm) Reference: left buccinator muscle (5 × 5 cm)	10, 50, 300 Hz, sham (2 mA, 5 min)	tACS during rest time between the first and two subsequent sessions of motor training (immediately and 30 min after tACS)	50-Hz tACS enhanced motor acquisition immediately after tACS, but this improvement disappeared 30 min after tACS
Hsu et al., 2019 [56]	Randomly assigned *n* = 59	Visuomotor multitask	Active: bilateral prefrontal (radius 1 cm) Reference: Afz, Fz, FCz (radius 1 cm)	6 Hz (in-phase), 6 Hz (anti-phase), sham (2 mA, 3 min × 4 sessions) Session interval: in-phase (1 min), anti-phase (5 min)	tACS during task	In-phase 6-Hz tACS enhanced multitasking performance, with an increase in posterior alpha and beta power. Anti-phase 6-Hz tACS had no effect
Berntsen et al., 2019 [55]	Randomly assigned *n* = 60	Bilateral hand motor sequence	Active: left M1 or left parietal or left prefrontal (3 × 3 cm) Reference: right frontopolar (3 × 3 cm)	Individual‘s alpha (M1, parietal, prefrontal), sham (1 mA, 20 min)	tACS during rest time between the first and second sessions of motor training	Individual‘s alpha-tACS over prefrontal enhanced motor acquisition compared with sham stimulation
Miyaguchi et al., 2020 [61]	Crossover *n* = 32	Bilateral pegboard task	Active: supplementary motor area (5 × 5 cm) Reference: left shoulder (5 × 5 cm)	20 Hz, 80 Hz, sham (1 mA, 2 min × 3 sessions) Session: random order of three stimulation conditions with 2-min intervals	tACS during task	Participants with higher initial motor performances showed greater motor acquisition during 20-Hz tACS, while participants with lower initial motor performances showed greater motor acquisition during 80-Hz tACS
Wessel et al., 2020 [63]	Crossover *n* = 15	Sequential grip force modulation task	Active: left cerebeller cortex (5 × 5 cm) Reference: left buccinator muscle (5 × 5 cm)	50 Hz, sham (2 mA, 20 min)	tACS during task	50-Hz tACS did not enhance motor acquisition during tACS or motor retention 24 hr after tACS
